# Inflammatory biomarker concentrations in dogs with gastric dilatation volvulus with and without 24-h intravenous lidocaine

**DOI:** 10.3389/fvets.2023.1287844

**Published:** 2024-01-04

**Authors:** Anna Brunner, Anna Lehmann, Bianca Hettlich, Laureen M. Peters, Camille Julie Doras, Katja-Nicole Adamik

**Affiliations:** ^1^Division of Small Animal Emergency and Critical Care, Department of Clinical Veterinary Medicine, Vetsuisse Faculty, University of Bern, Bern, Switzerland; ^2^Division of Small Animal Internal Medicine, Department of Clinical Veterinary Medicine, Vetsuisse Faculty, University of Bern, Bern, Switzerland; ^3^Division of Small Animal Surgery, Department of Clinical Veterinary Medicine, Vetsuisse Faculty, University of Bern, Bern, Switzerland; ^4^Clinical Diagnostic Laboratory, Department of Clinical Veterinary Medicine, Vetsuisse Faculty, University of Bern, Bern, Switzerland; ^5^Veterinary Public Health Institute, Vetsuisse Faculty, University of Bern, Bern, Switzerland

**Keywords:** canine, gastric torsion, cytokines, systemic inflammation, lidocaine

## Abstract

**Background:**

Canine gastric dilatation volvulus (GDV) is characterized by tissue ischemia, reperfusion, and systemic inflammation. Evidence exists that lidocaine exerts anti-inflammatory properties and potentially improves outcome.

**Design and setting:**

Prospective, randomized observational cohort study in client-owned dogs with GDV.

**Objective:**

The primary objective of the study was the determination of pro- and anti-inflammatory biomarker concentrations in dogs with GDV with and without intravenous (IV) lidocaine therapy. The second objective was the evaluation of side effects of lidocaine.

**Methods:**

Of 35 dogs included in the study, 20 dogs were assigned to receive lidocaine (LIDO) (2 mg/kg initially, followed by a continuous infusion at a rate of 50 μg/kg/min over 24 h) and 15 dogs not to receive lidocaine (NO-LIDO). Plasma concentrations of cytokines interleukin (IL)-6, IL-7, IL-8, IL-10, IL-15, IL-18, interferon gamma, keratinocyte chemotactic-like, monocyte chemotactic protein, and C-reactive protein (CRP) were measured at admission (prior any therapeutic intervention, T0), immediately after surgery (T1), at 24 h (T24), and at 48 h (T48) post-surgery.

**Results:**

No significant differences in concentrations of any cytokines were found between the LIDO- and the NO-LIDO group. Significant lower CRP concentrations (median [range]) were found in dogs with lidocaine compared to dogs without at T24 (97.5 pg/mL [46.3–161.7] vs. 127.9 pg/mL [26.9–182.0]; *p* = 0.046) and T48 (73.7 pg/mL [18.4–169.4] vs. 116.3 pg/mL [71.4–176.8]; *p* = 0.002). Dogs receiving lidocaine exhibited significantly impaired mentation, a prolonged period of anorexia, and longer hospitalization compared to dogs without lidocaine.

**Conclusion:**

Lidocaine administration had no impact on the plasma levels of cytokines during the 48-h study period, but significantly lower CRP concentrations were found at T24 and T48. Lidocaine’s potential side effects require careful decision making regarding its use.

## 1 Introduction

Gastric dilatation and volvulus (GDV) is a serious condition and characterized by gastric displacement, rapid intragastric gas accumulation, and an increase in intra-gastric pressure, leading to ischemia of the gastric wall with subsequent stomach wall necrosis. In addition, GDV may lead to a decrease in venous return and impairment of systemic circulation, resulting in obstructive shock and multiple organ damage. The goal of therapy is to improve the systemic circulation and gastric blood flow, by means of intravenous (IV) fluid resuscitation, gastric decompression, and surgical repositioning of the stomach (1–3).

Many of the complications associated with GDV are related to the reperfusion of previously ischemic areas, leading to ischemia–reperfusion injury (IRI) characterized by the production of reactive oxygen species (1, 4). The mechanisms behind the generation of reactive oxygen species have been well described in the literature (5, 6).

Previous studies have evaluated markers of inflammation and cell injury or necrosis in dogs with GDV. These studies found that plasma high mobility group box-1, cell-free DNA, procalcitonin, and C-reactive protein (CRP) were increased in GDV dogs, with procalcitonin at admission being predictive of non-survival (7). In addition, higher CRP concentrations were significantly linked with a negative outcome (8). In a study conducted among a subset of dogs with GDV included in the current research, a diverse spectrum of inflammatory patterns was detected. Specifically, interleukin (IL)-6, interferon (IFN)-γ, monocyte chemotactic protein (MCP)-1, IL-10, and CRP were predominantly linked to the inflammatory response, with the peak of this response occurring in the period between surgery and 24 h post-surgery (9).

Lidocaine is primarily recognized for its actions as a local anesthetic, an IV antiarrhythmic drug, and for its potential analgesic effect when administered IV (10–12). In addition, lidocaine has been recommended as a free radical scavenger and for the prevention of IRI (13, 14), including in dogs with GDV (1, 4, 15). Furthermore, lidocaine has several anti-inflammatory effects, the molecular mechanisms of which are poorly understood (13). No published study evaluated the effect of IV lidocaine on concentrations of different inflammatory markers in dogs with GDV.

The primary objective of the present study was to compare the concentrations of different plasma cytokines and CRP over a 48-h period and assess the potential anti-inflammatory effect in dogs with GDV who received a 24-h IV lidocaine therapy compared to those who did not. The hypothesis was that IV lidocaine therapy leads to lower concentrations of cytokines and CRP. As a secondary objective, the study also evaluated the potential side effects associated with lidocaine administration.

## 2 Materials and methods

### 2.1 Trial design

This study was a prospective, randomized, parallel-group, non-blinded cohort study conducted at a single center, involving client owned dogs with GDV. Dogs with GDV presented between June 2017 and September 2018 at the veterinary teaching hospital at the University of Bern, Switzerland were eligible for inclusion. The trial was approved by the Animal Experiment Committee of the Swiss Federal Veterinary Office (registration number BE 69/17), and informed owner consent was obtained for all dogs. The study adheres to the guidelines outlined in the standards of reporting trials in pets (PetSORT) statement (16).

### 2.2 Subjects

Data from some dogs in this study’s cohort were previously published in two other studies by the same institution (9, 17). Specifically, cytokine data from 15 dogs, referred to as the NO-LIDO group in this study, were reported in a prior study focusing on concentrations and kinetics (9, 17). As described (9), diagnosis of GDV was established by the presence of compatible clinical signs and was further confirmed by distinct radiographic findings, along with surgical intervention. Any dogs presenting with severe heart conduction blocks, weighing less than 15 kg, or those that were subjected to euthanasia due to financial reasons were excluded from the study.

### 2.3 Randomization

The enrolled dogs were randomly assigned to receive or not receive IV lidocaine for 24 h, using the permuted block technique with a block size of six subjects, consisting of three dogs receiving lidocaine and three dogs not receiving lidocaine. The assignment order was randomized by using sealed slips in an envelope.

### 2.4 Data collection

#### 2.4.1 Blood samples

The processing of blood samples followed the same protocol as previously described in a study conducted at the same institution (9). Briefly, blood samples were collected from affected dogs at four time points: at admission (prior to any therapeutic measures, T0), immediately post-surgery (approximately 5 min after completion of the last skin suture) (T1), 24 ± 4 h post-surgery (T24), and 48 ± 4 h post-surgery (T48). 1.3 mL K2-EDTA tubes and 9 mL heparin tubes (K2-EDTA and Li-Heparin LH/1.3, Sarstedt AG, Switzerland) were used for blood storage at T0, T24, and T48 for hematological (Advia^®^ 2120i, Siemens Healthcare Diagnostics AG, Switzerland) and biochemical (Cobas^®^ c501, Roche Diagnostics, Switzerland) analyses including CRP (Randox canine CRP, CP2798, Cobas^®^ c501, Roche Diagnostics, Switzerland) and lactate (RAPIDPoint^®^ 500; Siemens Healthcare AG, Switzerland). As described in the previous study, at T1 only 9 mL heparinized blood was collected, which was used for lactate analysis and then centrifuged, and of which an aliquot of 0.5 mL of plasma was then used for biochemical and CRP analysis (9). Remaining plasma of all timepoints was aliquoted and stored at −80°C within 1 h of blood collection, to be used later for batch analyses of cytokines. In case of intraoperative euthanasia, T1 blood sample was collected after gastric repositioning prior to euthanasia.

As described in the previous study, cytokines were analyzed using a Milliplex Canine Cytokine Panel, CCYTOMAG-90 K kit (Luminex MAGPIX analyzer, EMD Millipore, United States). Analysis was performed in duplicate of the following cytokines: IL-6, IL-7, IL-8, IL-10, IL-15, IL-18 (IFN-γ), keratinocyte chemotactic-like (KC-like), and (MCP-1), with samples randomized on each plate. Coefficients of variation (CV%) were computed for each sample’s replicate measurements. For samples exceeding detection limits, we did not repeat measurements with varying dilutions; instead, the maximum measurable value was employed. When cytokine concentrations fell below detectable levels, a result of “0” was recorded. All samples underwent overnight refrigerated incubation. Cytokine concentrations are reported in pg./mL (9).

#### 2.4.2 Illness severity scores

An acute patient physiologic and laboratory evaluation (APPLE_fast_) (18) score (including albumin, glucose, platelet count, blood lactate, and mentation score) was computed for each study dog upon admission.

The presence or absence of SIRS was assessed upon admission, and dogs were categorized as having SIRS if they met ≥2 of the following SIRS criteria defined by Hauptmann et al. (19): hypo- or hyperthermia (<38.1°C [100.6°F] or > 39.2°C [102.6°F]), tachycardia (heart rate > 120/min), tachypnea (respiratory rate > 20/min), leukocytosis (white blood cell [WBC] count >16 × 10^9^ cells/L [16 × 10^3^ cells/μL]) or leukopenia (WBC count <6 × 10^9^ cells/L [<6 × 10^3^ cells/μL]), and > 3% band neutrophils in the WBC count.

Dogs discharged from the hospital were defined as survivors, while dogs that naturally deceased or were euthanized due to a grave prognosis were categorized as non-survivors. For intraoperative euthanasia, IV pentobarbital (400 mg/kg given to effect; Euthasol^®^ 40% ad us. Vet, Virbac, Switzerland AG, 8152 Opfikon) was used.

#### 2.4.3 Evaluation of lidocaine side effects

Throughout the 48-h study period, parameters were assessed to evaluate the potential adverse effects of lidocaine. These encompassed cardiovascular parameters such as heart rate, systolic blood pressure (SunTech^®^ Vet20™ blood pressure monitor, Morrisville, NC, United States), and rectal temperature. Further, mentation, based on the APPLE_fast_ score, was also utilized, with scores ranging from 0 to 4 (18). A score of 0 represented normal mentation and ability to stand unassisted while a score of 4 indicated a dog unable to stand and react. Urination problems, such as unsuccessful urination despite assistance and an enlarged urinary bladder necessitating catheterization, were monitored during the post-surgical period. Additionally, duration of anorexia was evaluated by measuring the time (hours) until the dogs resumed eating within the 48-h post-surgical timeframe. Resumed eating was defined as eating at least 2–3 tablespoons of food at least 2 times per day.

### 2.5 Interventions

#### 2.5.1 General treatment protocol

A standard treatment protocol was implemented for all GDV dogs, as previously detailed in a prior study conducted by the same authors (9). To ensure cardiovascular stability, oxygen supplementation and IV fluid therapy (Plasma-Lyte A^®^, Baxter AG, Switzerland) was provided at the clinician’s discretion. Pain management was achieved through IV methadone (0.2 mg/kg; Methadon Streuli^®^, Streuli Pharma AG, Switzerland) or a fentanyl bolus (5 μg/kg; Fentanyl Curamed^®^, Actavis Switzerland AG, Switzerland) with subsequent continuous rate infusion (CRI) of fentanyl (5 μg/kg/h). In dogs with severe gastric distension, percutaneous gastrocentesis was performed using a 14- or 16-gauge needle once fluid therapy had commenced. Blood pressure was evaluated by oscillometric blood pressure measurement. Administered fluid volumes during initial stabilization, as well as in the intra-operative and post-operative period were documented.

#### 2.5.2 Lidocaine treatment protocol

The lidocaine treatment protocol followed the same protocol as previously described in a study conducted at the same institution (17). Briefly: dogs in the LIDO group received 2 mg/kg lidocaine (Lidokain 2% Streuli^®^, Streuli Pharma AG, 8730 Uznach, Switzerland) IV over 15 min along with IV fluid therapy but prior to any other treatments. This was followed by a lidocaine CRI (50 μg/kg/min) over 24 h, unless there was indication to discontinue (e.g., atrioventricular block) or extend treatment (e.g., sustained ventricular tachycardia). Dogs in the NO-LIDO group did not receive lidocaine during the study period, unless a medical reason developed, necessitating lidocaine administration (e.g., ventricular tachycardia with subsequent cardiovascular compromise). These dogs were excluded from the study.

#### 2.5.3 Anesthesia, surgery, and post-operative monitoring

Anesthesia, surgery, and post-operative monitoring followed the same protocol as previously described (9). Methadone (0.2 mg/kg, IV, Methadon Streuli^®^, Streuli Pharma AG, Switzerland) was used for premedication, and midazolam (0.2 mg/kg, IV; Dormicum^®^, Roche Pharma SA, Switzerland) and propofol (to effect; Propofol-^®^Lipuro, B. Braun Medical AG, Switzerland) were administered to induce anesthesia. After endotracheal intubation, anesthesia was maintained with isoflurane (titrated to effect; Isoflo^®^ ad us. vet., Zoetis GmbH, Switzerland) and oxygen (60–100%). Analgesia was provided by IV fentanyl (CRI at a rate of 5 μg/kg/h, Fentanyl Curamed^®^, Actavis Switzerland AG, Switzerland). Electrocardiogram (ECG), capnography, pulsoxymetry, arterial BP, and esophageal temperature were monitored throughout surgery.

Exploratory laparotomy was conducted by a board-certified surgeon or senior surgery resident and included decompression and repositioning of the stomach, followed by gastropexy. Gastric wall changes were classified as previously described and were based on gross appearance of the stomach after repositioning. Changes were divided into mild (no or slight red coloration of the stomach wall), moderate (purple or hemorrhaged gastric wall) and severe (green, gray or black gastric wall color and a friable and palpably thin gastric wall) (9). Heart rate and cardiac rhythm, mucous membrane color and capillary refill time, mentation, respiratory rate, rectal temperature, and oscillometric BP were monitored postoperatively. During the first 12–24 h postoperatively, continuous ECG was observed followed by intermittent ECG every 8 h until 48 ± 4 h postoperatively. For analgesia, dogs received IV fentanyl (CRI at a rate of 5 μg/kg/h, Fentanyl Curamed^®^, Actavis Switzerland AG, Switzerland) up to 24 h post-surgery, followed by IV buprenorphine (0.01–0.02 mg/kg q8h; Temgesic^®^, Indivior Schweiz AG). Additional treatment included IV isotonic crystalloids and omeprazole (1 mg/kg IV q12h; Omeprazol Streuli^®^, Streuli Pharma AG, Switzerland).

### 2.6 Statistical analyses

Statistical analyses were performed using MedCalc software (MedCalc^®^ Statistical Software version 22.007,^1^ 2023, Ostend, Belgium) and significance was set at *p* < 0.05 throughout. Some figures were made with NCSS 2023 statistical software [NCSS 2023 Statistical Software (2023); NCSS, LLC., Kaysville, Utah, United States].^2^ Shapiro–Wilk tests were used to assess normal distribution. As the majority of data were not normally distributed, all data are reported as median with range. Statistical differences of quantitative variables between groups (e.g., LIDO vs. NO-LIDO) were examined with Mann–Whitney rank sum tests. For each inflammatory markers studied, we analyzed the relationship between plasma concentration, treatment (LIDO vs. NO-LIDO) and kinetics (blood sampling time), adjusted by confounding factors, with a linear mixed effects model in R and the package lme4 (20). Time from admission to surgery, duration of anesthesia and duration of surgery were included as confounders. An interaction term between sampling time and treatment group was also added into the model to account for the kinetics of the inflammatory response. Outcome data (i.e., plasma concentrations of inflammatory markers) were square root transformed and assumptions of homoscedasticity and normality of residuals were assessed and met. The Akaike Information Criteria (AIC) was used to select the model that best fitted our data (21). Categorical variables among the groups were assessed using either Chi-squared tests or Fisher’s exact tests, as appropriate. Non-survivors were summarized in one group for statistical analysis.

## 3 Results

### 3.1 Cohort characteristics and outcome

**Figure 1:**
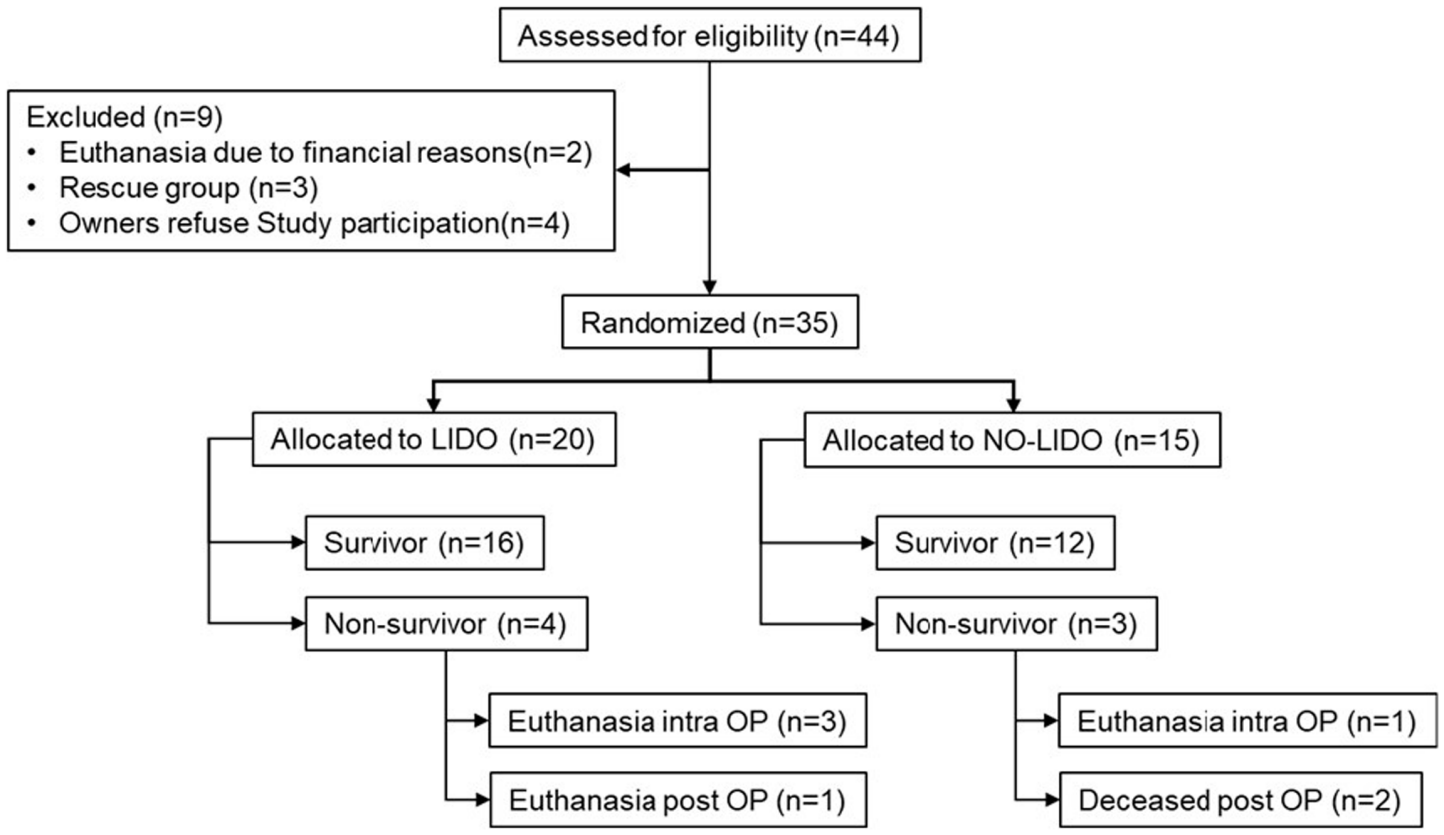
Inclusion and exclusion of 44 dogs presented with GDV into the study cohort. LIDO, GDV dogs treated with lidocaine; NO-LIDO, GDV dogs treated without lidocaine; rescue group, dogs initially enrolled in the NO-LIDO group but required IV lidocaine due to the development of clinically relevant ventricular arrhythmia; intra OP, intra-operatively, post OP, post-operatively.

Between June 2017 and September 2018, a total of 44 dogs with GDV were assessed for eligibility to be included in the study. Of these, two dogs were euthanized prior to surgery due to financial concerns and four dogs were not included due to missing owner consent. Of the 35 included dogs, 20 were randomly allocated to the LIDO group and 18 to the NO-LIDO group. Subsequently, three dogs from the NO-LIDO group needed to be excluded due to ventricular arrhythmia requiring lidocaine treatment, leaving a total of 15 dogs in the NO-LIDO group (Figure 1). Demographic, baseline and outcome data in dogs randomized to the LIDO or NO LIDO group are presented in Table 1. There was a total of 19 breeds. Following breeds were represented in the LIDO group: Great Dane (*n* = 5), German Shepherd (*n* = 3), Bernese Mountain Dog (*n* = 2), and one each of Border Collie, Briard, Dobermann, Dalmatian, Eurasian, Golden Retriever, Wirehaired Pointing Griffon, Irish Setter, Labrador Retriever, and Swiss Mountain Dog. In the NO-LIDO group, the following breeds were represented: Mixed breed dog (*n* = 5), Weimaraner (*n* = 2), St. Bernard (*n* = 2), and one each of Great Dane, Golden Retriever, Labrador Retriever, Spanish Mastiff, Newfoundland Dog, and Poodle.

**Table 1:** Demographic and baseline data (median, min-max) in dogs randomized to the LIDO or NO LIDO group.

Characteristic	LIDO (*n* = 20)	NO-LIDO (*n* = 15)
Age (years)	8.4 (1.5–13.6)	7.6 (2.1–14.5)
Sex (n)		
Female, intact	3	1
Female, neutered	2	6
Male, intact	5	4
Male, neutered	10	4
Body weight (kg)	37.7 (25.0–80.1)	34.9 (17.3–86.6)
Blood lactate (RI: 0.42–2.10 mmol/L)	3.12 (1.72–8.98)	2.77 (1.35–10.38)
APPLE_fast_ score	24 (15–33)	20 (10–34)

RI, reference interval.

GDV associated data and procedures in dogs randomized to the LIDO or NO LIDO group are presented in Table 2. No significant differences were observed between the two groups in terms of duration of clinical symptoms prior to admission, administered fluid volumes from admission until the end of surgery, and time between admission and the start of surgery. Duration of anesthesia and surgery was significantly longer in the LIDO group. During the course of the study, two dogs in the LIDO group and one dog in the NO-LIDO group developed ventricular arrhythmias, but no additional treatment was necessary. The overall mortality was 20% and no significant difference was found between the LIDO and NO-LIDO group (*p* = 1.0). In the LIDO-group, 4/20 dogs did not survive (Figure 1). Of these, euthanasia was performed intra-operatively in 3 dogs due to severe gastric wall changes, and early post-operatively in 1 dog due to hemoperitoneum and hypovolemic shock (owners declined further therapy). In the NO-LIDO group, 3/15 dogs did not survive. One dog was euthanized during surgery due to severe stomach wall changes, and two dogs (moderate and severe stomach changes each) suffered cardiac arrest (at 3 and 6 h after the surgery, respectively), which did not respond to cardiopulmonary resuscitation (Figure 1).

**Table 2:** GDV associated data and procedures (median, min-max) in dogs randomized to the LIDO or NO LIDO group.

Characteristic	LIDO (*n* = 20)	NO-LIDO (*n* = 15)	*P*-value
Duration of clinical signs prior to admission (min)	120 (60–540)	120 (60–360)	0.958
Transcutaneous gastrocentesis (n)	13	12	0.609
Time between admission and surgery (min)	102 (30–159)	88 (46–112)	0.115
Duration of anesthesia (min)	120 (70–180)	90 (60–195)	0.047
Duration of surgery (min)	85 (40–150)	65 (40–100)	0.038
Fluid volume (mL/kg) from admission to end of surgery	94 (59–207)	75 (52–217)	0.479
Gastric wall changes	Mild: 13	Mild: 10	0.999
	Moderate: 3	Moderate: 2	
	Severe: 4	Severe: 3	

### 3.2 Plasma cytokines and CRP concentrations

Mean coefficient of variation (CV) of duplicate measurements were 13%, with 11% of duplicates exceeding a CV of 25%, fulfilling the manufacturer’s quality guidelines. No values were above the limit of detection.

#### 3.2.1 LIDO vs. NO-LIDO group

**Figure 2:**
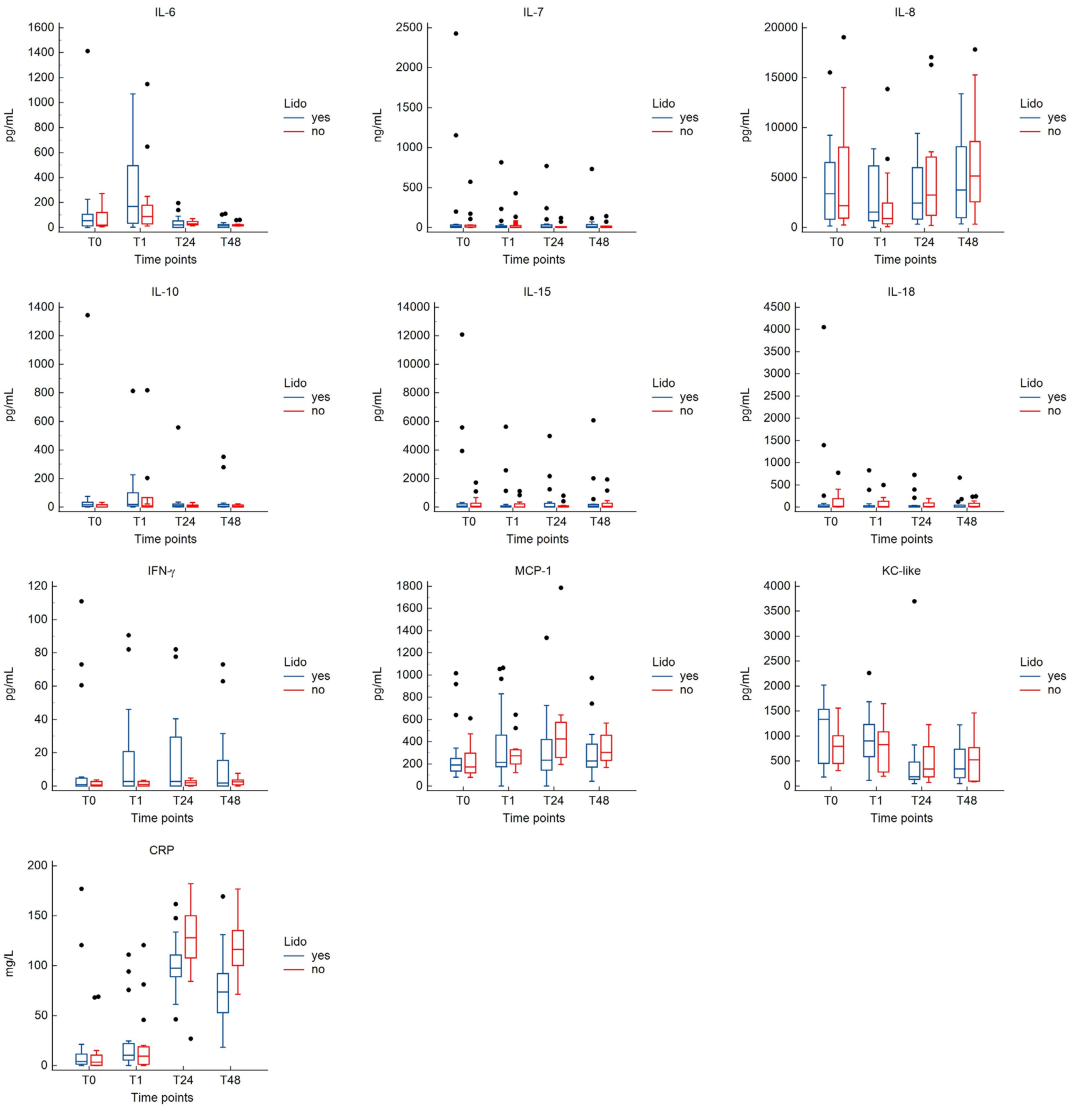
Box-and-whisker plots comparing cytokine and CRP concentrations between the LIDO and NO-LIDO groups across four measurement time points (T0, T1, T24, T48). Analytes include interleukine (IL)-6, IL-7, IL-8, IL-10, IL-15, IL-18, IFN-γ, KC-like, MCP-1, and CRP. The LIDO group is represented by blue bars (left) and the NO-LIDO group by red bars (right). The central box indicates the interquartile range (25th to 75th percentile) with the median represented by the midline. Outliers are depicted as individual dots.

No significant differences in cytokine concentrations were observed between the groups at any time point (Supplementary Table S1). Plasma concentrations[median (range)] of CRP were significantly lower in the LIDO group compared to the NO-LIDO group at T24 [97.5 pg/mL (46.3–161.7) vs. 127.9 pg/mL (26.9–182.0); *p* = 0.046] and T48 [73.7 pg/mL (18.4–169.4) vs. 116.3 pg/mL (71.4–176.8); *p* = 0.002] (Supplementary Table S1). The kinetics of cytokines and CRP in the LIDO and NO-LIDO group over the time period from T0 to T48 is illustrated in Figure 2.

The results from the linear mixed effects model are shown in Table 3. For some inflammatory markers, the sampling time significantly affected the measured plasma concentration. Compared to T0, T24, and T48 were associated with higher concentrations of CRP and MCP-1, but to a lesser extend in the LIDO group. Conversely, T24 and T48 were associated with a lower concentration of KC-like compared to baseline. T1 was associated with higher concentrations of IL6. For IL10, the plasma concentration is increased at T1 in both groups, but the magnitude of the increase is less in the LIDO group. However, the plasma concentration of IL10 is overall higher in the LIDO group than in the non-treated group. We found the same result for the plasma concentration of KC-like which is overall higher in the LIDO group, regardless of sampling time. IL8 concentration was significantly decreased at T1 and increased at T48, compared to T0. We observed that the time between admission and surgery significantly affected the plasma concentration of CRP, IL6, and MCP-1 toward a decrease and IL8 toward an increase (Table 3).

**Table 3:** Results of the mixed effects linear regression model analyzing the relationship between plasma concentration of inflammatory markers, treatment (LIDO vs. NO-LIDO) and kinetics (blood sampling time), adjusted by confounding factors.

Marker	Predictor	Category	*β*	Std. Errors	95% IC	*p*-value
IL6		(Intercept)	10.67	4.50	1.57, 19.93	0.018
	Group	LIDO	2.40	2.48	−2.53, 7.33	0.334
	Sampling time	T1	4.63	2.01	0.65, 8.62	0.021
		T24	−0.62	2.11	−4.90, 3.56	0.768
		T48	−1.92	2.11	−6.20, 2.26	0.363
	Admission-surgery time		−0.08	0.03	−0.15, −0.02	0.010
	Anesthesia duration		−0.02	0.04	−0.10, 0.06	0.637
	Surgery duration		0.08	0.05	−0.04, 0.19	0.158
	Group x Time	Lido x T1	−1.27	2.84	−6.91, 4.37	0.654
		Lido x T24	−2.75	2.91	−8.51, 3.12	0.346
		Lido x T48	−3.07	2.91	−8.83, 2.79	0.293
IL7		(Intercept)	2.59	6.00	−9.65, 14.84	0.666
	Group	LIDO	1.13	2.74	−4.38, 6.64	0.679
	Sampling time	T1	−1.00	1.46	−3.90, 1.91	0.495
		T24	−1.10	1.55	−4.22, 1.97	0.478
		T48	−0.91	1.55	−4.02, 2.16	0.559
	Admission-surgery time		0.00	0.04	−0.09, 0.09	0.929
	Anesthesia duration		−0.03	0.05	−0.14, 0.07	0.504
	Surgery duration		0.09	0.08	−0.06, 0.25	0.207
	Group x Time	Lido x T1	−2.09	2.07	−6.20, 2.02	0.313
		Lido x T24	−1.44	2.13	−5.66, 2.83	0.500
		Lido x T48	−2.00	2.13	−6.22, 2.27	0.349
IL8		(Intercept)	34.44	27.32	−21.08, 89.97	0.207
	Group	LIDO	−3.95	12.14	−28.40, 20.49	0.745
	Sampling time	T1	−18.37	5.74	−29.77, −6.97	0.001
		T24	4.57	6.10	−7.55, 16.67	0.454
		T48	12.90	6.10	0.78, 25.00	0.034
	Admission-surgery time		0.59	0.20	0.18, 1.00	0.004
	Anesthesia duration		−0.27	0.24	−0.76, 0.21	0.252
	Surgery duration		0.12	0.34	−0.57, 0.82	0.716
	Group x Time	Lido x T1	8.54	8.12	−7.58, 24.66	0.293
		Lido x T24	−15.01	8.38	−31.63, 1.63	0.073
		Lido x T48	−11.36	8.38	−27.98, 5.29	0.175
IL10		(Intercept)	2.57	6.86	−11.51, 16.68	0.708
	Group	LIDO	5.42	2.78	−0.19, 11.04	0.051
	Sampling time	T1	4.91	1.65	1.63, 8.20	0.003
		T24	0.62	1.83	−3.04, 4.27	0.735
		T48	0.33	1.83	−3.32, 3.98	0.855
	Admission-surgery time		−0.07	0.04	−0.17, 0.02	0.100
	Anesthesia duration		−0.04	0.05	−0.14, 0.07	0.462
	Surgery duration		0.14	0.07	−0.01, 0.29	0.053
	Group x Time	Lido x T1	−4.87	2.13	−9.12, −0.63	0.022
		Lido x T24	−3.31	2.27	−7.83, 1.23	0.146
		Lido x T48	−3.58	2.27	−8.11, 0.96	0.115
IL15		(Intercept)	−0.65	19.09	−39.49, 38.16	0.973
	Group	LIDO	6.52	8.32	−10.29, 23.32	0.434
	Sampling time	T1	−3.19	3.49	−10.12, 3.73	0.360
		T24	−2.98	3.71	−10.36, 4.37	0.421
		T48	1.46	3.71	−5.92, 8.81	0.694
	Admission-surgery time		0.02	0.14	−0.27, 0.31	0.901
	Anesthesia duration		−0.07	0.17	−0.41, 0.27	0.668
	Surgery duration		0.24	0.24	−0.24, 0.73	0.307
	Group x Time	Lido x T1	−3.36	4.93	−13.15, 6.43	0.496
		Lido x T24	−2.50	5.09	−12.59, 7.62	0.623
		Lido x T48	−6.65	5.09	−16.74, 3.47	0.191
IL18		(Intercept)	7.33	7.75	−8.46, 23.09	0.344
	Group	LIDO	−0.17	3.54	−7.30, 6.95	0.962
	Sampling time	T1	−2.16	1.91	−5.96, 1.64	0.259
		T24	−1.70	2.03	−5.75, 2.33	0.404
		T48	−1.48	2.03	−5.53, 2.55	0.467
	Admission-surgery time		−0.02	0.06	−0.14, 0.10	0.712
	Anesthesia duration		−0.08	0.07	−0.21, 0.06	0.259
	Surgery duration		0.15	0.10	−0.05, 0.34	0.126
	Group x Time	Lido x T1	−1.58	2.71	−6.96, 3.80	0.559
		Lido x T24	−1.02	2.79	−6.55, 4.54	0.714
		Lido x T48	−1.67	2.79	−7.20, 3.89	0.550
IFNγ		(Intercept)	−0.57	2.20	−5.04, 3.91	0.797
	Group	LIDO	0.62	0.95	−1.30, 2.54	0.513
	Sampling time	T1	−0.05	0.36	−0.77, 0.67	0.894
		T24	0.15	0.38	−0.62, 0.91	0.704
		T48	0.14	0.38	−0.62, 0.91	0.708
	Admission-surgery time		0.02	0.02	−0.01, 0.05	0.259
	Anesthesia duration		−0.01	0.02	−0.05, 0.03	0.472
	Surgery duration		0.02	0.03	−0.03, 0.08	0.407
	Group x Time	Lido x T1	0.78	0.51	−0.24, 1.79	0.128
		Lido x T24	0.03	0.53	−1.02, 1.07	0.957
		Lido x T48	−0.11	0.53	−1.16, 0.93	0.828
KC like		(Intercept)	35.09	7.81	19.23, 50.95	0.000
	Group	LIDO	7.72	3.91	−0.08, 15.53	0.048
	Sampling time	T1	0.07	2.72	−5.34, 5.48	0.979
		T24	−6.60	2.88	−12.30, −0.86	0.022
		T48	−6.28	2.88	−11.99, −0.55	0.029
	Admission-surgery time		−0.05	0.06	−0.16, 0.07	0.430
	Anesthesia duration		−0.07	0.07	−0.20, 0.07	0.330
	Surgery duration		0.05	0.10	−0.14, 0.25	0.593
	Group x Time	Lido x T1	−1.87	3.85	−9.52, 5.78	0.627
		Lido x T24	−6.85	3.96	−14.74, 1.01	0.084
		Lido x T48	−6.22	3.96	−14.11, 1.64	0.117
MCP1		(Intercept)	17.84	4.75	8.18, 27.52	0.000
	Group	LIDO	1.54	2.26	−2.99, 6.08	0.496
	Sampling time	T1	2.26	1.40	−0.53, 5.05	0.108
		T24	8.39	1.49	5.40, 11.33	0.000
		T48	4.42	1.49	1.44, 7.37	0.003
	Admission-surgery time		−0.07	0.03	−0.14, 0.00	0.039
	Anesthesia duration		−0.03	0.04	−0.11, 0.05	0.454
	Surgery duration		0.08	0.06	−0.04, 0.20	0.154
	Group x Time	Lido x T1	−1.46	1.99	−5.41, 2.48	0.462
		Lido x T24	−7.89	2.05	−11.94, −3.80	0.000
		Lido x T48	−4.10	2.05	−8.15, −0.01	0.045
CRP		(Intercept)	4.43	2.01	0.36, 8.55	0.028
	Group	LIDO	0.43	0.97	−1.50, 2.37	0.656
	Sampling time	T1	1.08	0.61	−0.13, 2.30	0.077
		T24	8.67	0.63	7.41, 9.91	0.000
		T48	8.41	0.65	7.12, 9.69	0.000
	Admission-surgery time		−0.03	0.01	−0.06, 0.00	0.036
	Anesthesia duration		0.00	0.02	−0.04, 0.03	0.881
	Surgery duration		0.02	0.02	−0.03, 0.07	0.448
	Group x Time	Lido x T1	−0.43	0.87	−2.15, 1.29	0.617
		Lido x T24	−1.51	0.88	−3.25, 0.25	0.086
		Lido x T48	−2.57	0.89	−4.34, −0.80	0.004

The reference categories are NO-LIDO and T0 for the variables Group and Sampling time respectively. x refers to the interaction term between these latter variables. IL, interleukin; IFN-γ, interferon gamma; KC-like, keratinocyte chemotactic-like; MCP-1, monocyte chemotactic protein; CRP, C-reactive protein.

#### 3.2.2 Survival vs. non-survival

**Figure 3:**
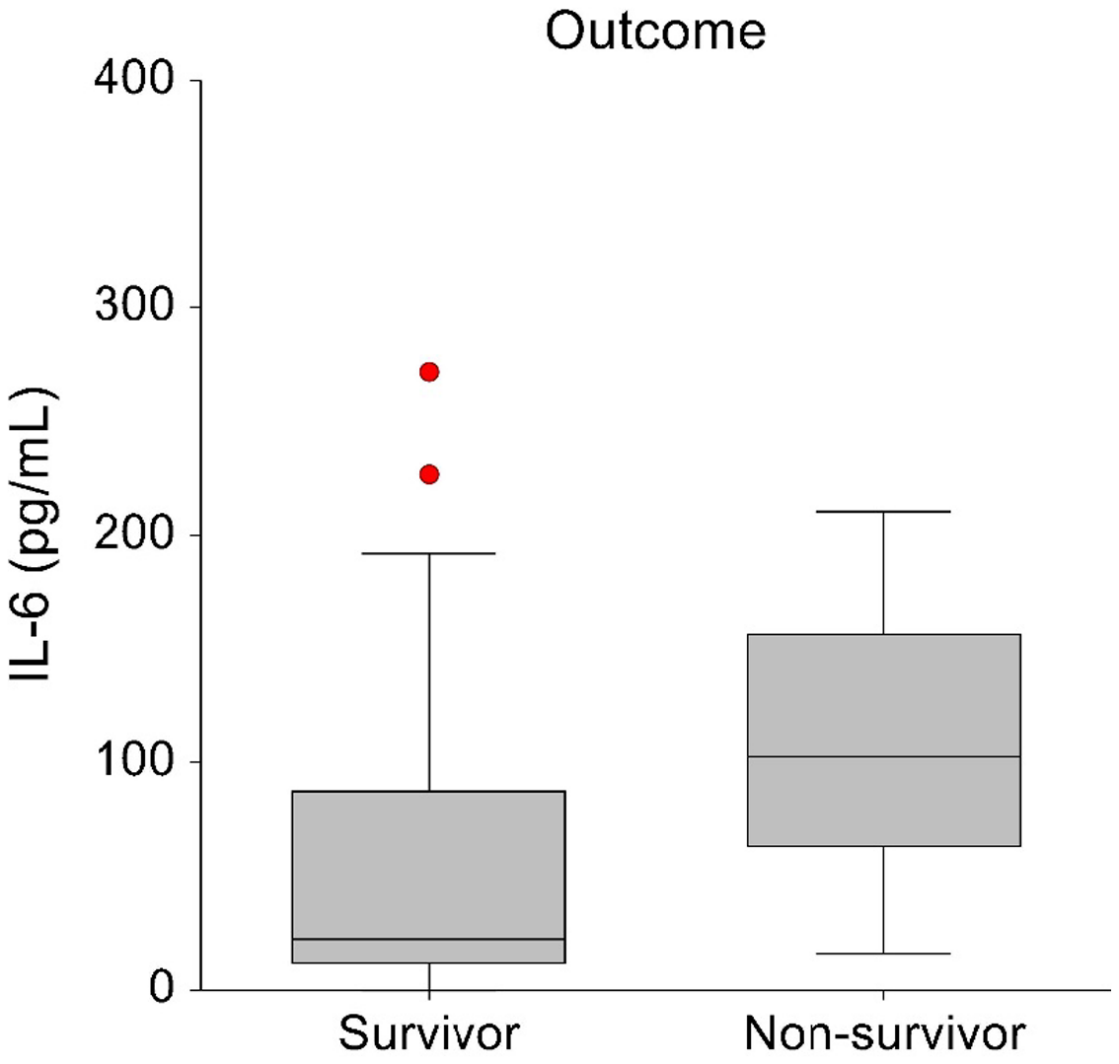
Box-and-whisker plots displaying the initial blood IL-6 concentrations in survivors (*n* = 28) and non-survivors (*n* = 7). The central box represents values within the lower to upper quartile range (25th to 75th percentile). To enhance visual clarity, one survivor with an IL-6 T0 concentration of 1413.84 pg/mL has been excluded from this graph.

Comparison of admission plasma cytokine concentrations between survivors and non-survivors revealed significantly higher IL-6 concentrations in non-survivors. Survivors had a median IL-6 concentration of 23 pg./mL (range, 0–1413.8 pg/mL) and non-survivor had a median IL-6 concentration of 102 pg/mL (range, 15.8–210.7 pg/mL; *p* = 0.043) (Figure 3).

### 3.3 Blood lactate concentrations

Admission blood lactate concentrations between the LIDO vs. the NO-LIDO group revealed no significant difference at any time point (Table 1).

### 3.4 APPLE_fast_ score and SIRS

At T0, 89% of dogs (31/35) were classified to have SIRS. No significant difference was found between the LIDO vs. NO-LIDO group. No significant difference in the admission APPLE_fast_ score between the LIDO and the NO-LIDO group was found (Table 1).

### 3.5 Adverse effects of lidocaine

**Figure 4:**
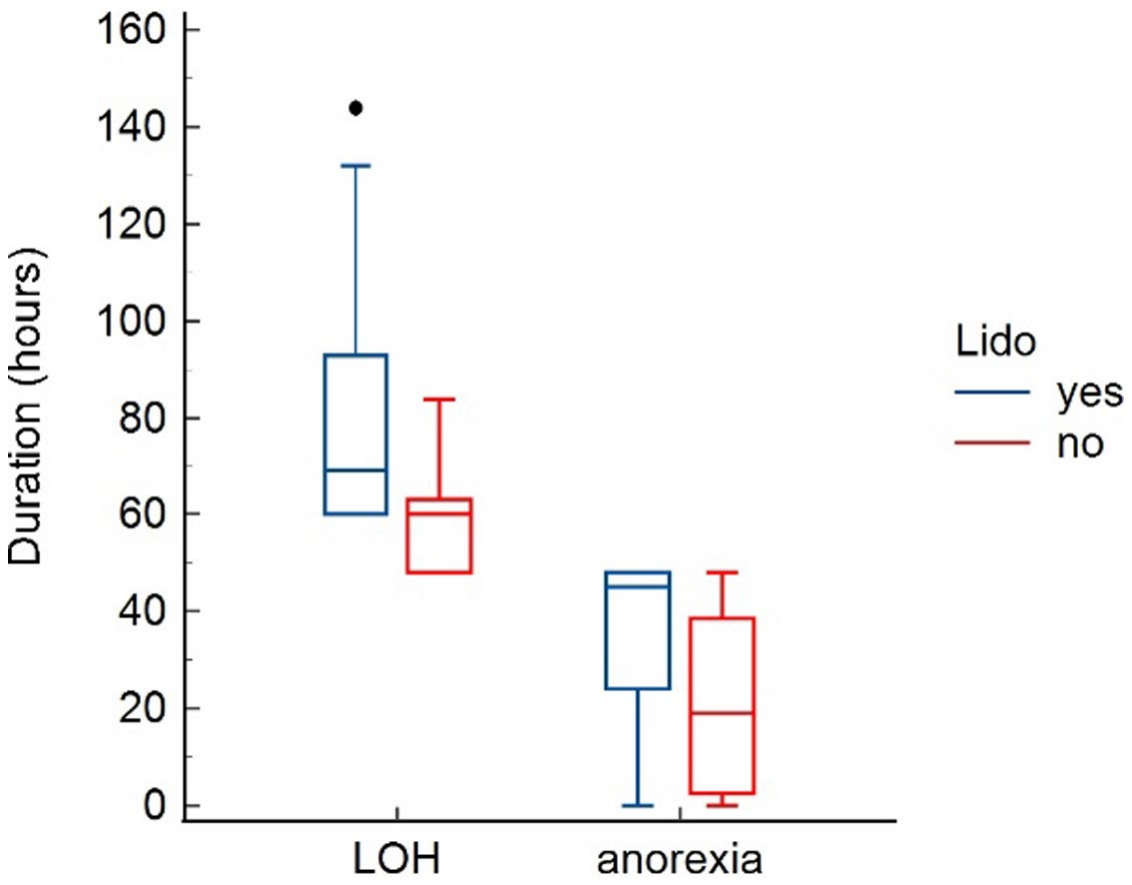
Box-and-whisker plots comparing length of hospitalization (LOH) and duration of anorexia between the LIDO and NO-LIDO group. The LIDO group is represented by blue bars (left) and the NO-LIDO group by red bars (right). The central box indicates the interquartile range (25th to 75th percentile) with the median represented by the midline. Outliers are depicted as individual dots.

Parameters assessed for potential adverse effects of lidocaine are presented in Table 4. No effects were found on heart rate and systolic blood pressure. Lidocaine was associated with a significant lower body temperature at T24 (*p* = 0.041). Further, dogs in the LIDO group revealed a significant prolonged duration of anorexia (*p* = 0.043), and a significant longer LOH (*p* = 0.013) (Figure 3). The mentation score in the LIDO group was consistently higher than in the NO-LIDO group at all assessed time points Regarding urination, 36.8% of male dogs (7/19) and 11.1% of female dogs (1/9) demonstrated urination problems during the postsurgical period. Specifically, 43.7% of dogs (7/16) in the LIDO group (6 males and 1 female) and 8.3% of dogs (1/12) in the NO-LIDO group (1 male) had difficulty urinating (*p* = 0.088) (Figure 4).

**Table 4:** Parameters assessed for potential adverse effects of lidocaine.

Variable	Time point	LIDO-group	NO-LIDO-group	*P*-value
Heart rate (bpm)	T_0_	132 (72–200)	140 (88–210)	0.828
	T_1_	80 (60–168)	94 (72–160)	0.202
	T_24_	80 (60–120)	82 (72–120)	0.429
	T_48_	73 (52–100)	80 (64–100)	0.181
Systolic blood pressure (mmHg)	T_0_	154 (99–198)	152 (103–194)	0.391
	T_1_	136 (85–218)	149 (84–182)	0.506
	T_24_	147 (125–183)	145 (120–222)	0.816
	T_48_	151 (104–197)	146 (118–194)	0.417
Rectal temperature (°C)	T_0_	38.4 (37.1–39.8)	38.4 (37.8–39.5)	0.652
	T_1_	37.2 (36.3–38.8)	37.4 (36.6–38.1)	0.555
	T_24_	37.7 (35.7–38.3)	38.0 (37.2–39.4)	0.041
	T_48_	37.9 (36.9–38.5)	38.1 (37.8–38.8)	0.075
Mentation score (0–4) (18)	T_0_	1 (0–3)	0 (0–3)	0.017
	T_24_	1 (0–3)	0 (0–2)	0.007
	T_48_	0 (0.3)	0 (0–0)	0.014
Duration of anorexia (h)	T_24_–T_48_	45 (0–48)	19 (0–48)	0.044
Urination problems (number of dogs, %)	T_24_-T_48_	7 (43.7%)	1 (8.3%)	0.088
LOH (days)	n/a	2.9 (2.5–6.0)	2.5 (2.0–3.5)	0.015

SBP, systolic blood pressure assessed with oscillometry (SunTech^®^ Vet20™ blood pressure monitor, Morrisville, NC, United States); Mentation score: 0, normal; 1, able to stand unassisted, responsive but dull; 2, can stand only when assisted, responsive but dull; 3, unable to stand, responsive; 4, unable to stand, unresponsive (18).

## 4 Discussion

Gastric dilatation volvulus is considered a classic example of non-infectious systemic inflammation, with IRI and presumed “hypercytokinemia” as major factors leading to the clinical picture of SIRS, organ damage and failure (3). In dogs with GDV, markers of cell damage and inflammation, such as cell free DNA, high-mobility group box-1, and procalcitonin were found to be significantly higher compared with healthy dogs (7). Higher procalcitonin concentrations at presentation were associated with nonsurvival (7) and a significant difference in high-mobility group box-1 between survivors and non-survivors over time was found (8). In a previous study in GDV dogs conducted at the same institution as this one, contrary to initial expectations, we observed increase in a limited number of pro-inflammatory cytokines (e.g., IL-6, IFN-γ, MCP-1) and IL-10 (9). The main focus of the present study was the determination of plasma concentrations of various inflammatory cytokines and CRP in dogs with GDV with and without a 24-h IV lidocaine CRI. The study aimed to investigate the potential anti-inflammatory effect of lidocaine in this context. Our results from comparison analyses indicate that lidocaine administration did not have any impact on the plasma levels of cytokines during the 48-h study period. However, dogs receiving lidocaine had significantly lower plasma levels of CRP at 24- and 48-h post-surgery. In the regression model, we found a significant interaction between the time effect of sampling and lidocaine treatment, in the sense of an anti-inflammatory effect, which means that the increase observed over time for CRP, IL10 and MCP-1 was diminished in the LIDO group. This is consistent with the results of comparison analyses for CRP. However, the regression model showed an adverse effect of lidocaine infusion on IL10 and KC-like overall concentrations.

The most severe complications associated with GDV arise from IRI and SIRS, leading to subsequent multiple organ failure. Ischemia reperfusion injury is a complex pathophysiological process involving various pathways and body systems (5, 6). Earlier studies involving experimental gastric dilation and GDV in dogs and cardiac ischemia in rats revealed the occurrence of necrotizing gastritis, cardiac dysfunction, and hepatocellular necrosis as consequences of IRI. The effectiveness of various treatments to combat IRI, including lidocaine, was evaluated (22–25). Besides the effect on IRI, lidocaine also exhibits anti-inflammatory effects by inhibiting leucocyte activation, adhesion, migration, and superoxide anion production, potentially attributed to its direct influence on macrophage and polymorphonuclear granulocyte functions (26). Its anti-inflammatory effects may be further attributed to the inhibition of interleukin release, a component of the inflammatory cascade (26). In a recent meta-analysis, encompassing 21 studies involving 1,254 patients and comparing the effectiveness of perioperative IV lidocaine to a placebo in individuals undergoing elective surgery, IV lidocaine demonstrated a significant reduction in the levels of various postoperative inflammatory cytokines and CRP (27). Several studies in the field of abdominal and colorectal surgeries in humans have shown that the perioperative administration of lidocaine has been effective in reducing the release of pro-inflammatory cytokines induced by the surgery, including IL-6 and IL-8 (27, 28). In animals, an inhibited production of inflammatory cytokines after lidocaine administration was demonstrated in horses (29), mice (30), and rabbits (31) with endotoxemia or septic peritonitis, respectively. Few clinical studies have been conducted in dogs to assess the anti-inflammatory and organ protective effects of IV lidocaine (4, 15, 17, 32). In a retrospective case series involving 75 dogs diagnosed with septic peritonitis, the administration of lidocaine (2 mg/kg, IV bolus, followed by a CRI of 50 μg/kg/min) during surgery was found to significantly enhance the likelihood of short-term survival following the surgical procedure. This improvement in survival was attributed to the anti-inflammatory effects of lidocaine (32). Bruchim et al. found that in dogs with GDV, IV lidocaine (2 mg/kg, IV bolus, followed by a CRI of 50 μg/kg/min) over 24 h reduces the risk for cardiac arrhythmias and kidney injury, which was attributed to the decrease in GDV-related IRI and its anti-inflammatory effects (4). In contrast, recent evaluation of renal biomarkers (e.g., neutrophil gelatinase-associated lipocalin) in dogs with GDV, conducted on the same GDV cohort as this study, did not show evidence of lidocaine-associated renoprotection (17). Findings of the present study also demonstrate that lidocaine administration did not result in a significant attenuation of cytokine expression, and there were no differences in mortality rates between the lidocaine and the control group. It is important to mention that, since both studies were conducted on almost the same dog cohort, a common underlying mechanism cannot be ruled out. The observed difference on concentrations of inflammatory cytokines after IV lidocaine between humans and dogs with GDV could be attributed to species differences or disease-specific variations, or both. Dogs and humans may have different physiological and immunological characteristics, possibly affecting the effectiveness of lidocaine as an anti-inflammatory drug. Further, GDV has unique inflammatory pathways that may not respond to lidocaine as in other conditions. Moreover, the current study only measured the effect on inflammatory markers, not on IRI. Nonetheless, we did observe significantly lower post-surgical CRP concentrations in dogs receiving lidocaine (9). C-reactive protein, a major acute phase protein in dogs, is recognized to elevate following surgery and its production is triggered by pro-inflammatory cytokines, such as IL-6 (33). Previous data indicate that in hyperacute conditions the serum CRP is normal (e.g., dogs with GDV and trauma) but increases during the initial hours of hospitalization (9, 34). Based on the difference in CRP concentrations between the two groups in our study, an anti-inflammatory effect of lidocaine can be assumed. Further research is necessary to elucidate the specific mechanisms and potential alternative cytokines involved in the anti-inflammatory action of lidocaine in GDV.

In the study by Bruchim et al., it was discovered that dogs administered a 24-h lidocaine CRI had a notably lower incidence of cardiac arrhythmias compared to those who did not (12% vs. 38%) (4). In the current investigation, the overall prevalence of ventricular arrhythmia was 16% which is comparably low (e.g., up to 42% in previous studies) (35, 36). Three dogs initially allocated to the NO-LIDO group needed to be excluded due to ventricular arrhythmia requiring lidocaine treatment. The authors of the current study could not statistically prove a cardioprotective effect of lidocaine-CRI, which is due to the low overall number of ventricular arrhythmias, but lidocaine probably prevented ventricular arrhythmias in the LIDO group.

A secondary objective of study was the evaluation of side effects of lidocaine. The use of lidocaine as part of multimodal analgesic strategies in the perioperative setting is controversial in terms of efficacy and safety (12, 37). In people, nausea, drowsiness, light-headedness, tinnitus and bradycardia were described as side effects after clinical doses (37). In dogs, depression, ataxia, muscle tremors, nausea, vomiting (usually transient) and cardiac effects are described (14, 38). In the present study, dogs in the LIDO group had a significantly longer duration of anorexia, impaired mentation, lower body temperature at T24, and a longer LOH. Nausea and anorexia are recognized side effects of lidocaine in dogs, and their occurrence is dependent on the dosage administered (39, 40). This undesirable side effect could most likely be resolved by dose reduction. In addition, the concomitant administration of anti-emetics should be considered in dogs receiving lidocaine CRI. In the present study, no anti-emetics were administered during the study period. The initial higher mentation score observed in the LIDO group prior to the initiation of lidocaine therapy makes the interpretation of the mentation scores at T24 and T48 challenging. It is possible that the LIDO group coincidentally was more sensitive to the sedative effect. However, at T24, a significant lethargy was noted in the LIDO group. A dose-dependent mild to moderate sedative effect of lidocaine in dogs is well described in the literature (38, 39, 41). The authors of the present study believe that the observed impairment in mentation was likely related to lidocaine. This sedative effect can pose a drawback, particularly during the post-operative phase of gastrointestinal surgery, as it may limit patients’ mobility, compromise their ability to protect their airways, and increase the risk of aspiration pneumonia. The authors have no explanation for the observed hypothermia at T24 and no plausible explanation was found in the literature (42). The hypothermia may be attributed to the depressive effect, although other cardiovascular parameters such as heart rate and blood pressure remaining within normal limits. Although if not significant, more dogs (mainly males) in the LIDO group had problems with urination (e.g., unsuccessful urination despite assistance and subsequently enlarged urinary bladder necessitating urinary catheterization). The authors speculate that depression and weakness associated with lidocaine treatment might be responsible for the inability to get up and go outside for urination. To ensure comfort of dogs receiving continuous IV lidocaine, it is essential to implement regular urinary bladder monitoring and perform urinary catheterization when necessary. The LOH was higher in the LIDO group, which was described previously (15). This is most probably a result of the described side effects of lidocaine in our study (anorexia, urine retention, and impaired mentation), which could have led to a delay in discharging the animals.

The current study has limitations. First, the lack of a blinded, placebo-controlled design in our study could introduce bias in various aspects, particularly since certain outcomes, such as adverse effects, are somewhat subjective. Although lidocaine is routinely used in the author’s institution’s treatment protocol for GDV, its inclusion may have prolonged the duration of anesthesia in the LIDO group and may have biased assessment of adverse effects. Utilizing a placebo control would have allowed for uniformity across all procedures. Further, we also observed the time between the admission and the surgery, and the duration of the surgery as the most important confounding factors influencing the fitting of the regression models. Indeed, the overall time taken for clinical management may substantially affect the laps between the sampling timepoint T0 (admission) and T1 (immediate post-surgery), as well as the kinetics of the inflammatory response. Therefore, the sampling time points may in fact not fully be comparable between dogs, especially T1. This difference should fade over time and affects to a lesser extent the interpretation of T24 and T48. The extended duration of anesthesia in the treated group could have diminished the potential beneficial effects of lidocaine. The effect of the time of the anesthesia was not found to be as important as the other times. Third, the exclusion of 3 dogs from the NO-LIDO group (due to clinically relevant ventricular arrhythmia) may introduce a potential bias that cannot be entirely ruled out. The precise cause of the arrhythmia in these dogs is not well understood, but it is plausible that the most severely affected dogs were excluded from the analysis. Fourth, although intraoperative euthanasia in four dogs was solely performed due to severe gastric wall changes and necrosis, we cannot completely rule out the possibility that euthanasia may have introduced bias to the outcome analysis of our study. There is a theoretical chance that some of these dogs might have survived if they were not euthanized. Finally, given the limited sample size, it’s important to acknowledge that the possibility of both type I and type II errors cannot be ruled out.

In conclusion, lidocaine administration did not have any impact on the plasma levels of cytokines during the 48-h study period, but significantly lower CRP concentrations were found at T24 and T48. Further data on this topic is required to definitively clarify whether lidocaine indeed has a specific anti-inflammatory effect. Dogs receiving lidocaine exhibited significantly impaired mentation, a prolonged period of anorexia, and longer hospitalization compared to dogs without lidocaine. The potential side effect must be carefully balanced against the presumed positive effects of lidocaine.

## Data availability statement

The raw data supporting the conclusions of this article will be made available by the authors, without undue reservation.

## Ethics statement

The animal studies were approved by the Animal Experiment Committee of the Swiss Federal Veterinary Office (registration number BE 69/17). The studies were conducted in accordance with the local legislation and institutional requirements. Written informed consent was obtained from the owners for the participation of their animals in this study.

## Author contributions

AB: Formal analysis, Investigation, Writing – original draft. AL: Writing – review & editing. BH: Writing – review & editing. LP: Writing – review & editing. K-NA: Writing – review & editing, Conceptualization, Formal analysis, Funding acquisition, Investigation, Methodology, Project administration, Supervision, Validation, Visualization, Writing – original draft. CD: Formal analysis, Writing – review & editing.

## Funding

The author(s) declare financial support was received for the research, authorship, and/or publication of this article. This study was financially supported by the Swiss Association for Small Animal Medicine, Lucretia Watkins, Hostig 6, CH-8132 Hinteregg and the Albert Heim Foundation, sekretariat@albert-heim-stiftung.ch (Project number: 133).

## Footnotes

^1^ https://www.medcalc.org

^2^ ncss.com/software/ncss
