# Supplementary figures and images for: Inflammatory biomarker concentrations in dogs with gastric dilatation volvulus with and without 24-h intravenous lidocaine
Source: Front Vet Sci. 2024 Jan 4;10:1287844. doi: 10.3389/fvets.2023.1287844 (PMC10794732; doi:10.3389/fvets.2023.1287844)

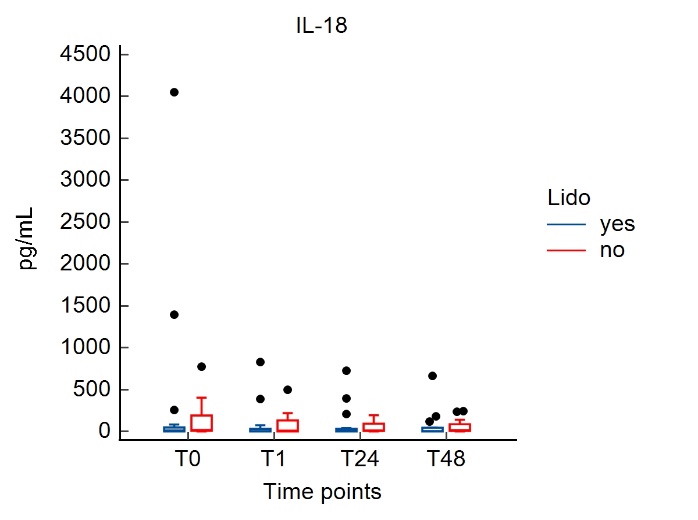

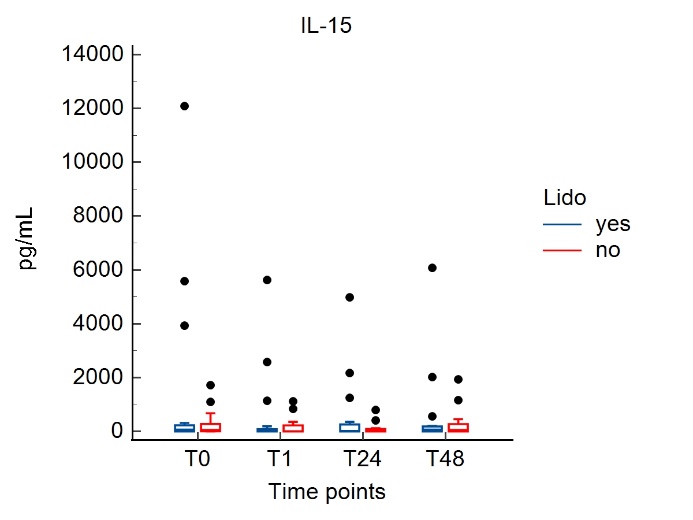

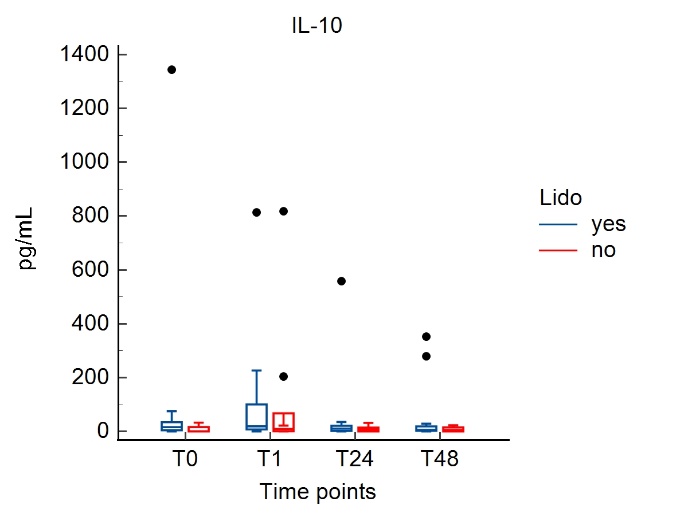

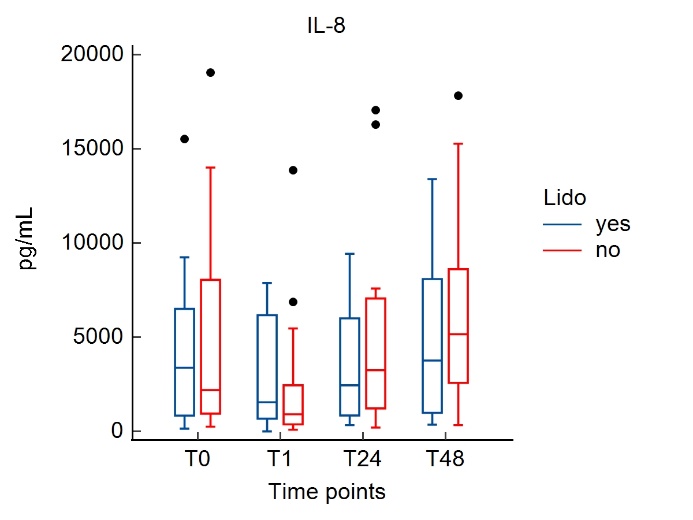

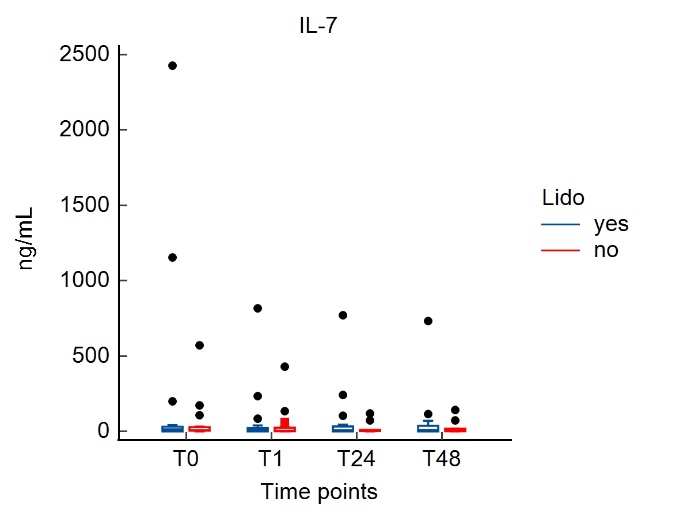

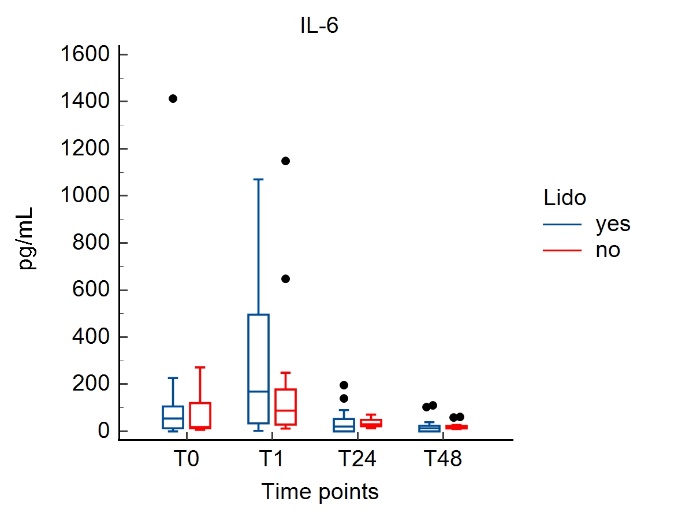


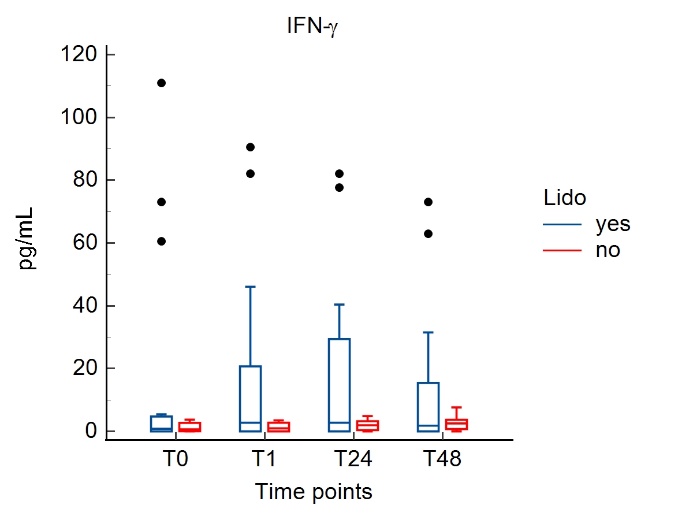

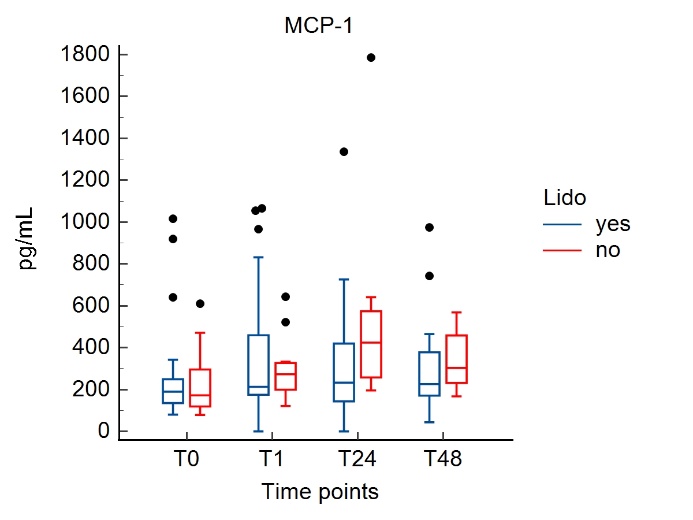

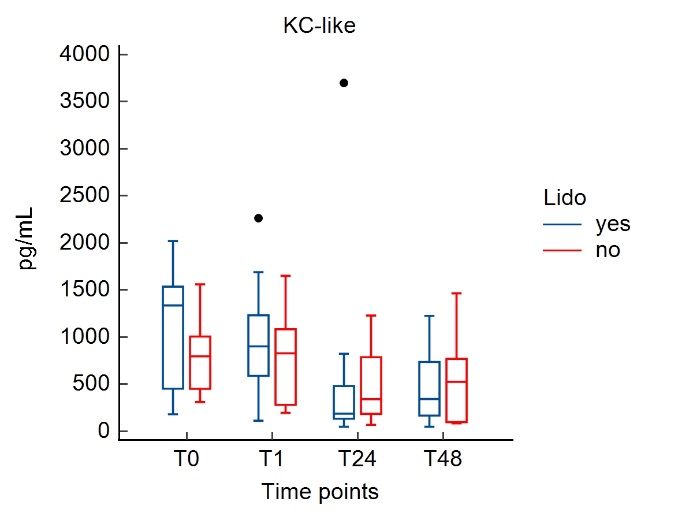


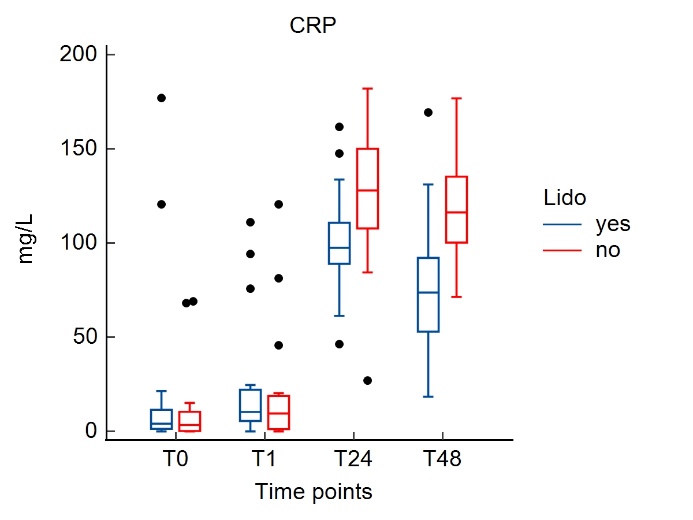

Supplement: Supplementary file 1 [file Data_Sheet_1.DOCX]
