# Supplementary material for: Inflammatory biomarker concentrations in dogs with gastric dilatation volvulus with and without 24-h intravenous lidocaine
Source: Front Vet Sci. 2024 Jan 4;10:1287844. doi: 10.3389/fvets.2023.1287844 (PMC10794732; doi:10.3389/fvets.2023.1287844)
Supplement: Supplementary file 2 [file Table_1.docx]

**Supplementary Table S1: Inflammatory markers (median, range) at admission (T0), post-surgery (T1), 24 hours (T24) and 48 hours (48) post-surgery in dogs with GDV with (LIDO-group) and without lidocaine (NO-LIDO-group).**

| **Variable** | **Time point** | **LIDO-group** | **NO-LIDO-group** | ***P-*value** |
| --- | --- | --- | --- | --- |
| IL-6  (pg/mL) | T_0_ | 54.6 (0-1413.8) | 18.6 (6.9-271.7) | 0.479 |
|  | T_1_ | 169.1 (2.1- 1070.3) | 88.0 (11.9-1149.1) | 0.515 |
|  | T_24_ | 20.4 (0-197.2) | 30.4 (13.1- 71.1) | 0.264 |
|  | T_48_ | 13.1 (0-110.5) | 16.6 (10.3-62.2) | 0.225 |
| IL-7  (pg/mL) | T_0_ | 14.0 (0-2426.2) | 7.3 (0-570.3) | 0.737 |
|  | T_1_ | 8.5 (0-815.1) | 3.8 (0-429.3) | 0.972 |
|  | T_24_ | 6.7 (0-770.8) | 4.6 (0-117.8) | 0.887 |
|  | T_48_ | 8.6 (0-732.0) | 5.7 (0-141.2) | 0.962 |
| IL-8  (pg/mL) | T_0_ | 3378.4 (146.7-15524.4) | 2192.8 (248.4-19064.2) | 0.755 |
|  | T_1_ | 1546.4 (0-7873.9) | 910.4 (89.1-13878.4) | 0.430 |
|  | T_24_ | 2444.3 (333.1-9429.4) | 3247.8 (202.4-17047.4) | 0.599 |
|  | T_48_ | 3760.2 (358.0-13392.2) | 5157.5 (332.2-17819.2) | 0.397 |
| IL-10  (pg/mL) | T_0_ | 15.9 (0-1343.9) | 0 (0-32.6) | 0.084 |
|  | T_1_ | 18.8 (0-814.3) | 8.5 (0-818.6) | 0.372 |
|  | T_24_ | 10.3 (0-557.5) | 6.9 (0-31.6) | 0.529 |
|  | T_48_ | 5.3 (0-351.9) | 4.8 (0-22.8) | 0.531 |
| IL-15  (pg/mL) | T_0_ | 64.3 (0-12098.5) | 50.5 (0-1707.3) | 0.711 |
|  | T_1_ | 33.3 (0-5618.7) | 0.0 (0-1119.4) | 0.826 |
|  | T_24_ | 16.6 (0-4984.0) | 12.1 (0-800.9) | 0.769 |
|  | T_48_ | 67.0 (0-6080.9) | 50.5 (0-1922.2) | 0.886 |
| IL-18  (pg/mL) | T_0_ | 10.7 (0-4054.6) | 16.0 (0-773.2) | 0.304 |
|  | T_1_ | 3.7 (0-829.5) | 9.8 (0-497.5) | 0.529 |
|  | T_24_ | 4.9 (0-725.7) | 11.5 (0-192.6) | 0.491 |
|  | T_48_ | 3.1 (0-662.3) | 11.6 (0-240.3) | 0.367 |
| IFN-γ  (pg/mL) | T_0_ | 0.8 (0-111.1) | 0.6 (0-3.7) | 0.836 |
|  | T_1_ | 2.8 (0-90.5) | 1.0 (0-3.5) | 0.164 |
|  | T_24_ | 2.8 (0-82.1) | 2.0 (0-4.9) | 0.708 |
|  | T_48_ | 1.8 (0-73.1) | 2.5 (0-7.6) | 0.541 |
| KC-like  (pg/mL) | T_0_ | 1337.4 (181.7-2021.3) | 796.6 (311.2-1561.2) | 0.122 |
|  | T_1_ | 902.9 (113.2-2266.3) | 829.3 (196.0-1652.1) | 0.372 |
|  | T_24_ | 187.6 (49.3-3700.0) | 341.9 (70.1-1230.4) | 0.423 |
|  | T_48_ | 343.9 (48.8-1226.11) | 525.9 (85.0-1465.4) | 0.873 |
| MCP-1  (pg/mL) | T_0_ | 190.0 (79.9-1017.1) | 172.2 (78.5-610.2) | 0.805 |
|  | T_1_ | 212.9 (0-1066.3) | 273.0 (121.1-642.3) | 0.607 |
|  | T_24_ | 232.5 (0-1336.1) | 424.1 (195.6-1786.4) | 0.121 |
|  | T_48_ | 225.9 (43.4-974.5) | 303.1 (167.2-568.3) | 0.241 |
| CRP  (RI:0-10.7 mg/L) | T_0_ | 4.1 (0-177.0) | 3.4 (0-68.9) | 0.559 |
|  | T_1_ | 10.3 (0-110.9) | 9.5 (0-120.4) | 0.435 |
|  | T_24_ | 97.5 (46.3-161.7) | 127.9 (26.9-182.0) | 0.046 |
|  | T_48_ | 73.7 (18.4-169.4) | 116.3 (71.4-176.8) | 0.002 |

IL, interleukin; IFN-γ, interferon gamma; KC-like, keratinocyte chemotactic-like; MCP-1, monocyte chemotactic protein; CRP, C-reactive protein; RI, reference interval; P-value, between group comparison. For cytokine concentrations in normal dogs the reader is referred to reference Brunner et al.^9^.
